# Current State of Temporal Bone Education: National Survey of U.S. Otolaryngology Residency Programs

**DOI:** 10.1002/lary.70545

**Published:** 2026-04-02

**Authors:** Adam Karkoutli, Daniel E. Bestourous, Mana Espahbodi, Neil S. Patel, Matthew L. Carlson, Richard K. Gurgel

**Affiliations:** ^1^ Department of Otolaryngology‐Head and Neck Surgery University of Utah Salt Lake City Utah USA; ^2^ Department of Otolaryngology‐Head and Neck Surgery Mayo Clinic Rochester Minnesota USA

**Keywords:** medical education, otology/neurotology, resident education

## Abstract

**Objectives:**

To characterize the structure, resources, and educational practices of temporal bone laboratory training across ACGME‐accredited U.S. otolaryngology residency programs.

**Methods:**

A national cross‐sectional survey was distributed to program directors and faculty responsible for temporal bone education. Survey domains included curriculum structure, training frequency, faculty involvement, assessment practices, and access to laboratory resources. Descriptive statistics and univariable regression analyses were performed to explore associations between program characteristics and neurotology fellowship pursuit.

**Results:**

Thirty‐seven programs responded (28.2%). Laboratory structure varied widely, including longitudinal (monthly or weekly) and condensed (annual or 2–3 courses/year) formats. Programs with longitudinal sessions reported more annual lab hours than condensed formats (median 42 vs. 16 h/year, *p* = 0.003). All programs had access to cadaveric temporal bones (mean 2.25 ± 1.13 bones per resident/year), though five reported fewer bones than residents. The mean drill‐to‐resident ratio was 0.59, with 10.8% reporting a 1:1 ratio. Formal performance evaluation was used by 43.2% of programs. On exploratory univariable analysis, longer otology rotation duration was associated with having at least one neurotology fellow within five years (*p* = 0.025). Larger resident cohort size (*p* = 0.048) and older laboratory instruments (*p* = 0.043) were associated with producing more than one fellow.

**Conclusion:**

There is substantial variability in temporal bone education across U.S. otolaryngology training programs. Programs differ in curricula, resources, and access to cadaveric and simulation‐based training. Standardization of core components may improve educational equity and training consistency.

**Level of Evidence:**

N/A.

## Graphical abstract

Temporal bone education across U.S. otolaryngology residency programs varies widely in curriculum structure, resource allocation, and evaluation methods. Longitudinal training models are associated with greater annual laboratory exposure compared to condensed formats. Standardizing core components of temporal bone training may improve equity and readiness for otologic surgery.

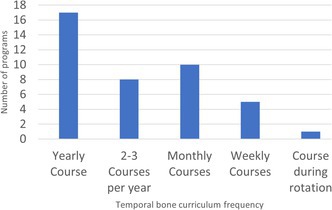

## 1 Introduction

Temporal bone laboratory education is a cornerstone of otolaryngology residency training and represents the initial exposure that most trainees have to otologic surgery. Mastery of temporal bone anatomy is essential for safe and effective surgical intervention in the lateral skull base and middle ear, where the proximity of critical neurovascular structures demands high precision.

The ACGME requires residents to develop anatomical knowledge through cadaveric dissection, temporal bone laboratory training, or surgical simulation [1]. These settings provide a low‐risk environment for building spatial understanding, mastering complex temporal bone anatomy, and refining technical skills before clinical practice. Accordingly, temporal bone education is essential for operative competency and patient safety, reducing risks such as facial nerve injury, hearing loss, and intracranial complications [2, 3, 4]. This philosophy reflects a foundational principle of surgical education articulated nearly a century ago by Dr. William Worrall Mayo: “There is no excuse today for the surgeon to learn on the patient” [5]. This tenet underscores the critical importance of cadaveric and simulation‐based training, ensuring that residents develop technical proficiency in a controlled, mentored environment before operating on live patients.

Despite its importance, no national curriculum exists for temporal bone education, and training varies widely across residency programs. Although the ACGME recognizes its role in procedural competency, it provides limited guidance on frequency, curriculum design, or assessment methods [1]. This lack of standards has resulted in substantial variability in educational models, from structured longitudinal curricula to single‐session bootcamps. Inequities in access to faculty mentorship, cadaveric specimens, equipment, and simulation resources further amplify these differences. Prior studies suggest that even among programs meeting minimum case requirements, resident exposure to otologic surgery remains inconsistent [6].

Multiple studies have documented wide variation in residents' self‐reported comfort with otologic surgery, even among senior trainees [6, 7]. Unlike many areas of otolaryngology, where surgical skills overlap through shared soft tissue techniques, otology requires specialized expertise in precision bone drilling and microscopic manipulation that does not readily transfer from other subspecialties. In the authors' opinion, this distinction makes otology one of the most challenging areas for senior residents to achieve sufficient competency for independent practice. Although minimum case requirements may be met, many residents report limited independent experience with key otologic procedures and concerns about readiness for unsupervised practice. This variability has been partly attributed to differences in access to high‐quality temporal bone training. Prior work has shown that greater participation in cadaveric dissection correlates with improved performance on standardized mastoidectomy assessments, underscoring the value of hands‐on training [8]. These findings highlight a gap between procedural volume and surgical preparedness. They suggest that educational structure, rather than case quantity alone, plays a critical role in competency development.

Given these concerns, a clearer understanding of how temporal bone education is delivered across otolaryngology programs is needed. To our knowledge, no national survey has comprehensively examined variation in curricula, resources, instructional methods, or faculty involvement. This study addresses this gap by surveying faculty in ACGME‐accredited otolaryngology programs to characterize current temporal bone training practices and identify opportunities for standardization.

## 2 Methods

### 2.1 Study Design

This was a cross‐sectional survey study conducted to assess the current state of temporal bone laboratory education among ACGME‐accredited otolaryngology residency programs in the United States.

This study was deemed “Non‐human subjects research” and therefore exempt from review by the University of Utah Institutional Review Board (IRB #00177044).

### 2.2 Survey Development

A 22‐item questionnaire was developed by the study team in consultation with otolaryngology education faculty (see Supporting Information S1). The survey included a mix of multiple‐choice, Likert scale, and short‐answer questions, designed to assess key domains of temporal bone education. These domains included are as follows:

- Availability and structure of cadaveric dissection labs
- Frequency and duration of lab sessions
- Faculty involvement and supervision
- Access to equipment (e.g., drills, microscopes)
- Use of simulation‐based training (e.g., 3D models, virtual reality)
- Resident assessment and feedback
- Neurotology fellowship pursuit and match outcomes.

### 2.3 Participants and Distribution

The survey was distributed electronically to program directors, neurotology faculty, and/or designated educational leads at all ACGME‐accredited otolaryngology residency programs in the United States, from 9/24/2024 to 10/14/2024. One reminder email was sent to bolster the response rate.

### 2.4 Data Collection and Analysis

Survey responses were collected anonymously using REDCap. Data were summarized using descriptive statistics. Programs were categorized by neurotology fellowship presence and by fellowship production within the prior five years. Univariable logistic regression was used to evaluate binary outcomes (≥ 1 fellow and > 1 fellows within 5 years) and univariable negative binomial regression for fellowship counts. Multivariable modeling was not performed due to limited sample size and outcome events. All analyses were conducted using IBM SPSS Statistics, version 27 (IBM Corp., Armonk, NY).

## 3 Results

### 3.1 Respondent Characteristics

**FIGURE 1:**
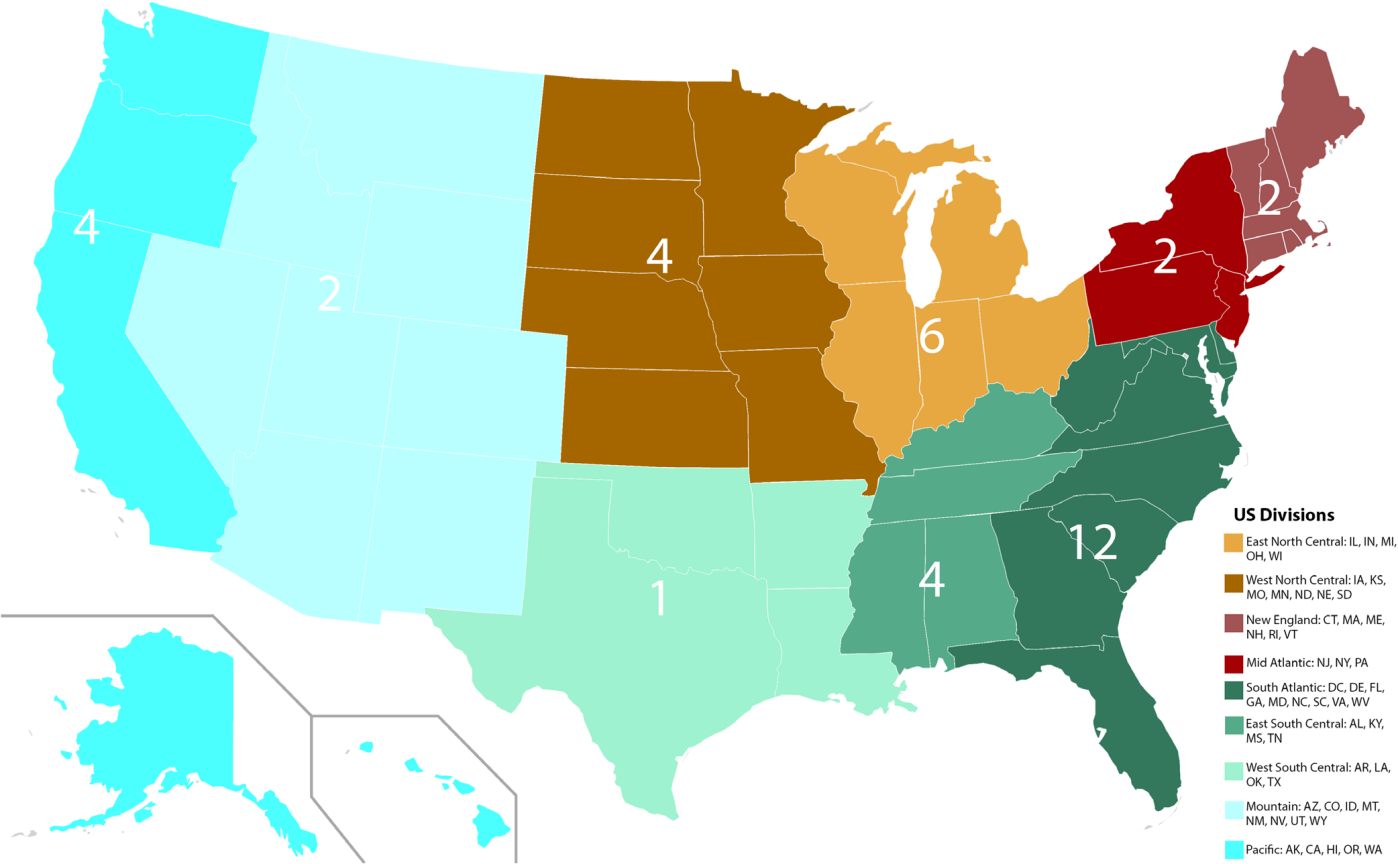
Number of Responding Otolaryngology Residency Programs by U.S. Census Division.

A total of 37 responses were obtained from the 131 accredited otolaryngology programs in the United States (28.2%). These programs were geographically diverse and included both university‐based and community‐based institutions. Among the responding programs, 14 (37.8%) reported affiliation with an ACGME‐accredited neurotology fellowship and an additional 3 programs had non‐accredited otology fellowships. Program size across surveyed residency programs had a median of 16 residents (range 10–30). The regional distribution of responding programs is illustrated in Figure 1.

### 3.2 Temporal Bone Laboratory Curriculum

**FIGURE 2:**
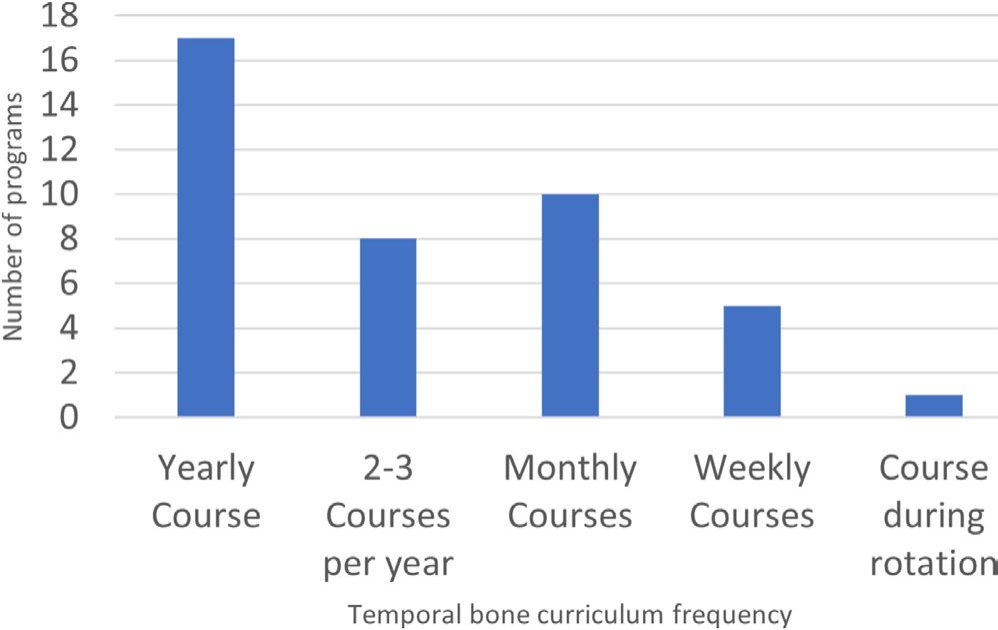
Frequency of temporal bone curriculum.

Across programs, curricular scheduling varied: 12 programs (32%) offered monthly dissection sessions, 6 (16%) offered weekly sessions, 10 (27%) concentrated training into a single annual course, 10 (27%) offered 2–3 courses per year, and 1 program (3%) provided dissection only during a dedicated otology rotation. Programs could select multiple options (Figure 2). On average, programs with condensed formats (annual and 2–3 courses per year) reported a median of 16.0 h of training per year, while those with monthly or weekly sessions reported a median of 42.0 h annually (*p* = 0.003).

**FIGURE 3:**
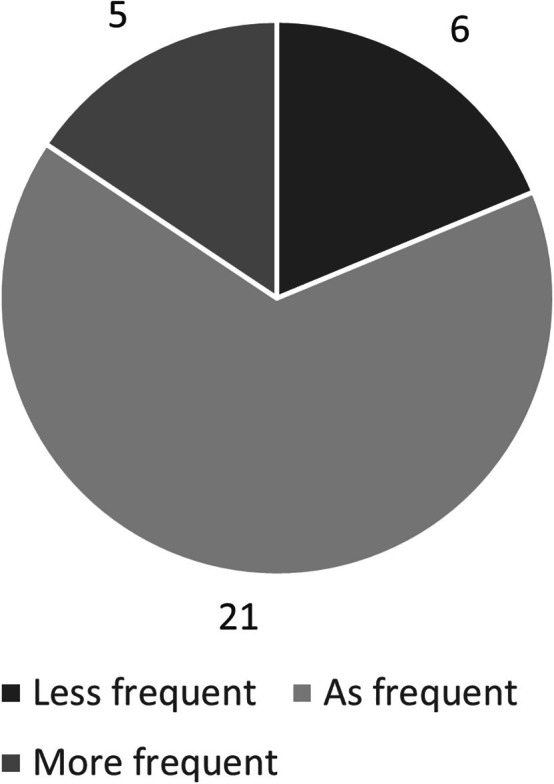
Relative Frequency of Temporal Bone Didactic Sessions Compared to Temporal Drilling Lab.

Many programs reported incorporating didactic instruction alongside dissection‐based training. A total of 32 programs (86.5%) included paired didactics. On average, residents received 2.30 ± 0.35 h of didactic instruction per month. Over half of respondents (53.1%) reported using structured source material, most commonly (*n* = 8) the *Otologic Surgery* textbook [9]. Regarding frequency, the majority of programs offered didactics at a rate equal to lab sessions, while others provided them more or less frequently (Figure 3).

### 3.3 Faculty Involvement and Supervision

Oversight of the temporal bone curriculum was most commonly provided by a combination of faculty neurotologists and other surgical faculty, reported in 20 programs, while 17 programs reported that oversight was provided exclusively by faculty neurotologists. The average number of faculty neurotologists per program was 2.8 (range 1–8).

Of the 14 programs with neurotology fellows, fellows were frequently involved in teaching; 8 programs reported that fellows served as instructors alongside faculty, and another 8 programs indicated that fellows acted as both instructors and participants. In contrast, only 1 program reported no fellow involvement in the temporal bone lab.

### 3.4 Access to Resources and Facilities

Access to temporal bone lab resources and facilities varied widely across surveyed programs. All programs (100%) reported access to human cadaveric temporal bones, though the quantity and distribution per resident differed. On average, programs used 40.57 temporal bones per year (median 31.5; range 8–120), corresponding to 2.25 ± 1.13 bones per resident (median 2.0; range 0.53–6.0). However, five programs reported having fewer temporal bones than residents.

A minority of programs (13.5%) supplemented cadaveric training with 3D‐printed synthetic temporal bones. In addition to core dissection infrastructure, programs reported use of various technological adjuncts to enhance temporal bone education. The most commonly available tools included endoscopes (*n* = 23) and closed‐circuit TV systems (*n* = 17). Fewer programs incorporated virtual atlases (*n* = 7), 3D printed models (*n* = 4), 3D anatomy didactics (*n* = 4), virtual drilling simulators (*n* = 1), or exoscopes (*n* = 1).

Programs reported an average of 9.83 drilling stations, with a mean drill‐to‐resident ratio of 0.59. Only 4 programs (10.8%) had as many drilling stations as residents. Most programs reported a deficit, with an average shortfall of nine drilling stations relative to total resident count. The age of temporal bone laboratories varied, with a median lab age of 11 years (IQR: 5.5–19.5 years). Instrument age was relatively modern, with 75.7% of programs reporting that the majority of their equipment was 10 years old or less.

Over half of programs (55.3%) reported resident participation in external cadaveric dissection courses, which provided additional access to specimens, instrumentation, and expert faculty. However, 30.4% of programs did not offer financial reimbursement for these courses.

### 3.5 Assessment and Evaluation

Formal performance assessment during temporal bone dissection was reported by 43.2% of programs (*n* = 16). Among these, the majority (81.3%, *n* = 13) used self‐devised rubrics, while 18.8% used evaluation tools developed externally. Despite this, more than half of programs (*n* = 21) reported having no formal evaluation method in place. Fourteen programs (37.8%) reported providing awards for performance in the temporal bone lab. These included monetary awards, non‐monetary recognition, or a combination of both.

### 3.6 Exploratory Analysis: Program Characteristics and Neurotology Fellowship Pursuit

#### 3.6.1 Predictors of ≥ 1 Neurotology Fellowship Pursuit (Univariable Analysis)

On univariable logistic regression analysis, the total number of dedicated otology rotation months was significantly associated with having at least one resident pursue neurotology fellowship within the last 5 years (odds ratio [OR], 1.49; 95% confidence interval [CI], 1.05–2.11; *p* = 0.025).

Faculty‐to‐resident ratio, total resident cohort size, temporal bone drill hours per month, temporal bone stations per resident, bones per resident, curriculum structure (condensed vs. longitudinal), didactics frequency, instrument age, and the postgraduate year at which residents first drill in the lab were not significantly associated with fellowship pursuit (all *p* > 0.05).

#### 3.6.2 Predictors of > 1 Neurotology Fellowship Pursuit (Univariable Analysis)

On univariable logistic regression analysis, larger resident cohort size was significantly associated with producing more than one neurotology fellow within the past 5 years (OR 1.16; 95% CI, 1.00–1.35; *p* = 0.048). Instrument age was also associated with fellowship production (OR 2.15; 95% CI, 1.03–4.52; *p* = 0.043). Programs reporting older temporal bone instruments were more likely to have produced more than one neurotology fellow.

All other factors, including faculty‐to‐resident ratio, otology rotation duration, drilling time, access to stations and specimens, curriculum structure, and timing of initial drilling, were not significantly associated with producing more than one fellow (all *p* > 0.05).

#### 3.6.3 Predictors of Neurotology Fellowship Count (Negative Binomial Regression)

Negative binomial regression was used to model fellowship counts. On univariable analysis, no curriculum or resource variables were significantly associated with the number of neurotology fellows produced within five years (all *p* > 0.05), including faculty‐to‐resident ratio, stations per resident, bones per resident, otology rotation duration, drilling hours, didactics frequency, and curriculum structure.

## 4 Discussion

Overall, this survey revealed broad variability in faculty involvement, lab infrastructure, resource access, and evaluation practices across U.S. otolaryngology programs.

This study highlights significant heterogeneity in temporal bone lab resources, teaching structures, and evaluation methods across U.S. otolaryngology programs. Disparities in access to dissection materials, equipment, and standardized feedback mechanisms may directly influence training experience and confidence.

This variability echoes historical concerns about medical education standardization. In 1910, Abraham Flexner published a report that described the state of medical education in the United States at that time. Based on his extensive travels and rigorous observations of medical schools, Flexner described wide variability in the quality of medical education in the United States. As he reported that there was, “no other country in the world in which there is so great a distance and so fatal a difference between the best, the average, and the worst” schools [10]. Although this study does not carry the historical impact of the Flexner report, it underscores the need to periodically evaluate national educational standards. This national survey of ACGME‐accredited otolaryngology residency programs demonstrates substantial variability in the structure, frequency, and resources of temporal bone laboratory education in the United States.

Our findings align with prior literature highlighting variability in resident comfort with otologic procedures and suggest that educational exposure, rather than case volume alone, may play a more important role in shaping surgical confidence [6, 7]. Prior studies have highlighted the value of cadaveric dissection in skill development. For example, Mowry et al. demonstrated that increased participation in cadaveric temporal bone dissection was associated with significantly improved performance on standardized mastoidectomy assessments [8]. These findings underscore the educational benefit of hands‐on dissection opportunities, especially when paired with structured oversight and dedicated curricular time.

### 4.1 Faculty and Fellow Involvement

Oversight of temporal bone curricula was most commonly shared between neurotologists and other surgical faculty (*n* = 20), while 17 programs reported exclusive neurotologist oversight. Among programs with fellows (*n* = 17), fellows participated in teaching in 16 programs, with only one program reporting no fellow involvement. The extent and quality of teaching involvement were not assessed.

### 4.2 Access to Resources and Facilities

Although all programs reported access to cadaveric temporal bones, limited complementary resources likely constrain hands‐on training. Mismatches between resident numbers and drilling stations reduce simultaneous dissection, repetition, autonomy, and individualized feedback. Even with adequate specimens, insufficient infrastructure may limit effective resource use and educational yield.

Limited adoption of supplemental training modalities further highlights variability in resource allocation. Only a minority of programs used 3D‐printed temporal bones, despite their potential to augment cadaveric dissection, particularly in settings with limited specimens or high resident‐to‐resource ratios.

Although more than half of programs supported external dissection courses, limited financial reimbursement may restrict access and exacerbate training inequities. These findings suggest that disparities in infrastructure and institutional support, rather than specimen availability alone, shape the quality and consistency of temporal bone education across programs.

### 4.3 Assessment and Evaluation

The limited use of structured performance assessment highlights a gap in temporal bone education. The lack of standardized, validated evaluation tools limits objective assessment of technical skills and comparability across programs, with feedback often relying on subjective observation. Broader adoption of competency‐based assessment frameworks may improve consistency in skill evaluation and support progression toward independent otologic practice.

Recognition programs were relatively common, with 37.8% of programs offering awards for performance in the temporal bone lab. While awards can reinforce motivation and highlight achievement, they are not substitutes for structured assessment tools.

### 4.4 Exploratory Analysis: Fellowship Pursuit

In exploratory analyses, longer dedicated otology rotation duration was associated with producing at least one neurotology fellow within five years, while larger program size was associated with producing more than one fellow. Instrument age was also associated with fellowship production, though this likely reflects institutional longevity or established mentorship structures rather than equipment quality itself. Notably, laboratory‐specific variables such as drill hours, station availability, and bones per resident were not independently associated with fellowship pursuit. Together, these findings suggest that sustained clinical exposure and broader institutional culture may influence subspecialty interest more than isolated laboratory metrics.

### 4.5 Institutional Temporal Bone Laboratory Training Model

At the University of Utah Department of Otolaryngology Head and Neck Surgery, residents participate in a structured four‐year temporal bone laboratory curriculum spanning PGY2 through PGY5. This institutional model is presented as an illustrative example and is not intended to represent a recommended or standardized approach.

Residents are excused from clinical duties to participate in monthly, faculty‐led temporal bone laboratories with additional instruction and support from the neurotology fellow. The curriculum is organized around the O*tologic Surgery* textbook, with two textbook chapters assigned and covered each month in parallel with corresponding laboratory objectives (see Supporting Information S2 for an overview of the institutional temporal bone curriculum) [9].

Each session includes predefined drilling assignments aligned with didactic content and focused on stepwise skill development. Monthly sessions are preceded by a brief quiz and a one‐hour lecture. Residents drill at least two temporal bones longitudinally each year, with a third reserved for an end‐of‐year practicum examination. The curriculum is supplemented by an annual full‐day temporal bone laboratory with a guest speaker.

Resident performance is assessed annually using a composite score based on monthly quizzes, de‐identified grading of a year‐long drilled temporal bone using a modified Welling scale [11], and anonymous evaluation of the practicum temporal bone. Composite scores are calculated for each resident, and the top‐performing resident is recognized with a monetary award at graduation.

## 5 Limitations

This study has several limitations. The overall survey response rate was modest (28.2%). Programs affiliated with ACGME‐accredited neurotology fellowships were disproportionately represented among respondents. Of 30 ACGME‐accredited neurotology fellowship programs nationally at the time of the survey, 14 responded (46.7%), compared with 23 of 101 non‐fellowship programs (22.8%). This imbalance may bias results toward better‐resourced institutions with more established otologic infrastructure and may overestimate national access to temporal bone resources. Self‐reported data may introduce recall bias, and faculty responses may not fully reflect resident experience. The absence of resident‐level outcome measures limits the ability to directly link curricular structure and resources to trainee performance. Future studies should incorporate resident feedback, objective competency assessments, and multi‐institutional benchmarking to better evaluate training variability and outcomes.

## 6 Future Directions

These findings highlight the need for greater standardization in temporal bone education. Defining core curricular expectations for training frequency, procedural objectives, and assessment methods, along with broader adoption of validated evaluation tools and simulation‐based training, may reduce disparities across programs. Future studies should evaluate whether training differences influence surgical competency or neurotology fellowship pursuit.

## 7 Conclusion

Temporal bone laboratory education is essential in otolaryngology training, yet its delivery varies widely across ACGME‐accredited programs. This study identifies differences in curriculum structure, resources, faculty involvement, and assessment practices that may affect resident preparedness for otologic surgery. Given the technical complexity of otologic procedures, standardizing core elements of temporal bone education may improve training quality and equity. These findings highlight the need for collaborative efforts to define best practices and develop national guidelines for otologic training.

## Funding

The authors have nothing to report.

## Conflicts of Interest

The authors declare no conflicts of interest.

## Supporting information


**Supporting Information: 1**: Temporal bone education survey distributed to U.S. otolaryngology residency programs.

**Supporting Information: 2**: Illustrative example of the University of Utah institutional temporal bone laboratory curriculum.

## Data Availability

The data that support the findings of this study are available from the corresponding author upon reasonable request.
