# Supplementary material for: Current State of Temporal Bone Education: National Survey of U.S. Otolaryngology Residency Programs
Source: Laryngoscope. 2026 Apr 2;136(9):3966–72. doi: 10.1002/lary.70545 (PMC13460714; doi:10.1002/lary.70545)
Supplement: Supplementary file 1 [file LARY-136-3966-s001.pdf]

Temporal Bone Lab Education in United States

Temporal bone lab education is an integral part of Otolaryngology resident training aimed at increasing competency in otologic and lateral skull base surgery. The purpose of this survey is to compile information about the variability in temporal bone education across the United States in order to describe the current state of temporal bone education of Otolaryngology Residency Programs. Responses will be used as a part of a study conducted by the University of Utah Department of Otolaryngology - Head and Neck Surgery.

This survey is intended to be completed by Otolaryngology Residency Program Directors or Otology and Neurotology Fellowship Directors, and/or faculty members overseeing a program's temporal bone education. Please complete only one survey per program. By completing this anonymous survey, you are consenting to the use of this data for the purposes of this study. This study was deemed "Non-human subjects research" and therefore exempt from review by the University of Utah Institutional Review Board (IRB #00177044).

We greatly appreciate your time and contribution to this study!

What regional division of the United States is your program located in?

- ◯ Northeast - New England (CT, ME, MA, NH, RI, VT)
- ◯ Northeast - Middle Atlantic (NY, NJ, PA)
- ◯ Midwest - East North Central (IN, IL, MI, OH, WI)
- ◯ Midwest - West North Central (IA, KS, MN, MO, NE, ND, SD
- ◯ South - South Atlantic (DE, DC, FL, GA, MD, NC, SC, VA, WV)
- ◯ South - East South Central (AL, KY, MS, TN)
- ◯ South - West South Central (AR, LA, OK, TX)
- ◯ West - Mountain West (AZ, CO, ID, NM, MO, UT, NV, WY)
- ◯ West - Pacific West (AK, CA, HI, OR, WA)

(Based on the United States Census Bureau)

What is the total number of residents in your program?

____

Does your institution have an ACGME-accredited Neurotology fellowship?

- ◯ Yes
- ◯ No

Does your program offer a non-accredited Otology fellowship?

- ◯ Yes
- ◯ No

How many fellowship-trained Neurotologists does your program employ?

____

Does your program have a dedicated Otology & Neurotology rotation for residents?

- ◯ Yes
- ◯ No

How many total months of operative and clinical Otology and Neurotology training do residents at your program receive over 5 years?

____

At what PGY level do residents get their first operative experience with a Neurotologist or Otologist?

- ◯ PGY1
- ◯ PGY2
- ◯ PGY3
- ◯ PGY4
- ◯ PGY5
- ◯ None of the above

In the last 5 years, how many of your program's graduating residents have pursued additional otologic training in the form of either a Neurotology or Otology fellowship?

____

radio button?

Does your program offer a dissection-based temporal bone education course for residents?	◯ Yes<br>◯ No, but a prosection course course is offered<br>◯ Neither a dissection nor a prosection course is offered<br>(A dissection-based education course refers specifically to a hands-on resident experience in which residents use tools and techniques employed in otologic and skull base surgery to study temporal bone anatomy and surgery.)
Does your program offer a didactics-based temporal bone education course for residents?	◯ Yes<br>◯ No
Is your temporal bone lab curriculum:	☐ Concentrated into a single course each year<br>☐ Concentrated into 2-3 courses each year<br>☐ Spread out into monthly courses<br>☐ Spread out into weekly courses<br>☐ Given only during residents' dedicated otology rotations
Is your temporal bone education overseen by:	◯ Faculty neurotologist(s), only<br>◯ Faculty neurotologist(s) and other faculty or non-faculty surgeons<br>◯ Faculty surgeon(s) that do not include a fellowship-trained neurotologist<br>◯ Non-faculty neurotologist(s), with or without other faculty or non-faculty surgeons<br>◯ Non-faculty surgeon(s) that do not include a fellowship-trained neurotologist<br>◯ Other
Please explain what other individuals oversee your program's temporal bone education:	__________________________________
How many stations are in your temporal bone lab?	__________________________________
At what PGY level do residents begin drilling in temporal bone lab?	◯ PGY1<br>◯ PGY2<br>◯ PGY3<br>◯ PGY4<br>◯ PGY5
How many hours per month are spent in the temporal bone lab?	__________________________________
How many hours per year is your program's temporal bone course?	__________________________________
To what extent do Neurotology fellows at your program participate in temporal bone dissection?	◯ Provide sole instruction to residents in dissection course<br>◯ Provide instruction to residents in dissection course alongside faculty<br>◯ Participate in dissection course alongside residents<br>◯ Both participate in dissection alongside residents and provide instruction to residents<br>◯ None of the above

To what extent do Otology fellows at your program participate in temporal bone dissection?	◯ Provide sole instruction to residents in dissection course<br>◯ Provide instruction to residents in dissection course alongside faculty<br>◯ Participate in dissection course alongside residents<br>◯ Both participate in dissection alongside residents and provide instruction to residents<br>◯ None of the above
Does your temporal bone dissection course follow a handbook, such as the House Institute Temporal Bone Dissection Manual by Dr. Ralph Nelson or a similar resource?	◯ Yes<br>◯ No
What dissection manual or handbook does your dissection course utilize?	__________________________________
What is the source of your temporal bones?	☐ Human cadaveric donor<br>☐ Animal cadaver<br>☐ 3D Printed<br>☐ Virtual
How are your temporal bones obtained?	☐ Pre-harvested cadaveric temporal bones donated to program or purchased by program<br>☐ Harvested from cadavers donated directly to program or purchased by program<br>☐ Harvested from cadavers donated to or purchased by medical school or other department<br>☐ Harvested from cadavers donated to or purchased by third party (medical device company, e.g.)<br>☐ None of the above
Who oversees and performs the harvest of cadaveric temporal bones used for your dissection course?	☐ Otolaryngology Program or Department at your home institution<br>☐ Another program or department at your home institution<br>☐ Otolaryngology Program or Department from another institution<br>☐ Another program or department from another institution<br>☐ A third party vendor or service (medical device company, e.g.)
Are whole head specimens available, or available by request, for performing more advanced procedures and approaches?	◯ Yes, and the curriculum incorporates a dedicated dissection for these approaches<br>◯ Yes, but there is no dedicated dissection in the curriculum for these approaches<br>◯ No, whole head specimens are not available<br>◯ Unsure
Does your program have access to fresh or lightly embalmed specimens?	◯ Yes, and the curriculum has dedicated dissections (i.e. soft tissue work) where these are used<br>◯ Yes, but the curriculum does not have dedicated dissections where these are used<br>◯ No, only fully fixed specimens are available<br>◯ Unsure
How many total temporal bones per year are used by your residents?	__________________________________

Are specimens readily available upon request or is there a delay in a resident's ability to acquire a temporal bone? Select the best answer.	◯ Specimens are readily available upon request<br>◯ Specimens are available within 3 months of a request<br>◯ Specimens are available within 6 months of a request<br>◯ Specimens are available within 12 months of a request<br>◯ Specimens are available and guaranteed, but delay may exceed 12 months<br>◯ Specimen availability is not guaranteed<br>◯ Unsure of availability
Which of the following best describes the laboratory used for dissection courses?	◯ Lab at home institution managed by Otolaryngology Department/Division and used exclusively for temporal bone dissections<br>◯ Lab at home institution managed by Otolaryngology Department/Division and used for all otolaryngology dissections<br>◯ Lab at home institution managed by another department and used only for otolaryngology dissections<br>◯ Lab at home institution shared by multiple departments<br>◯ Lab at a different institution<br>◯ Lab at a third-party institution (medical device company, e.g.)
Approximately what year was the lab used for your dissection course built?	__________________________________<br>(If you are unsure, please enter "N/A".)
Which of the following best describes how drills and instruments are obtained for used in your dissection course?	☐ Purchased by the Otolaryngology Program or Department<br>☐ Purchased by another department or program<br>☐ Purchased by Department of Graduate Medical Education<br>☐ Purchased by trainees<br>☐ Donated by the hospital or another entity within your institution<br>☐ Donated by an outside institution, hospital, or third party (medical device company, e.g.)<br>☐ None of the above<br>(Select all that apply)
Are drills, instruments, and equipment used for other dissection courses besides temporal bone?	◯ No, equipment is only used in temporal bone lab<br>◯ Yes, equipment is shared for other otolaryngology dissection courses<br>◯ Yes, equipment is shared with other departments for their individual dissection courses
Approximately how new is the equipment used in your temporal bone lab?	◯ Less than 5 years old<br>◯ 5-10 years old<br>◯ 11-15 years old<br>◯ 15-20 years old<br>◯ Greater than 20 years old

Do residents have access to prosthetics (PORPs, TORPs, cochlear implant electrodes, etc) for use in the dissection lab?

- ◯ Yes, as part of a dedicated lesson(s) within the temporal bone lab curriculum
- ◯ Yes, but there is no dedicated lesson(s) within the temporal bone lab curriculum and these are instead used for independent study or practice
- ◯ No, prosthetics are not available or must be obtained independently by residents

What types of technology, if any, do you use to augment your temporal bone education?

- ☐ Virtual atlas
- ☐ Virtual drilling simulators
- ☐ 3D printing
- ☐ 3D anatomy didactics
- ☐ Endoscopes
- ☐ Closed TV sets
- ☐ Other
- ☐ None

Please explain what other technology is used by your program to augment temporal bone education.

____

Do any of your residents attend an external temporal bone course?

- ◯ Yes
- ◯ No

An “external” temporal bone course would include any couse offered outside of your home institution, either by another institution or an industry sponsor

Is attendance to an external temporal bone course mandatory for residents at your program?

- ◯ Yes
- ◯ No

What percentage of your residents attend an external temporal bone course?

- ◯ 0-20%
- ◯ 21-40%
- ◯ 41-60%
- ◯ 61-80%
- ◯ 81%-100%

Is resident attendance to an external temporal bone courses subsidized or funded by your program?

- ◯ Yes, the program covers all expenses for residents that attend external courses
- ◯ Yes, the program partially covers some expenses for residents that attend external courses
- ◯ Yes, residents can apply for grants or scholarships to cover some or all of the costs of external courses
- ◯ No, but residents are offered other non-monetary incentives for attending external courses
- ◯ No, residents must pay out of pocket for external courses and are not offered other incentives

Does your program have a way of formally assessing resident temporal bone drilling performance?

- ◯ Yes
- ◯ No

How is resident performance assessed?

- ◯ Rubric or grading scale developed by own program
- ◯ Rubric or grading scale developed by another program/individual
- ◯ Other

Please explain how resident performance is assessed

____

Does your program give an award or prize for the top performing resident(s)?	◯ Yes, monetary award<br>◯ Yes, non-monetary award<br>◯ Yes, both a monetary and non-monetary award<br>◯ Yes, recognition without an award<br>◯ No
Is your temporal bone dissection paired with a didactic lecture?	◯ Yes<br>◯ No
Does your didactics course utilize source material, such as an otologic textbook?	◯ Yes<br>◯ No
What source material does your didactics course utilize?	__________________________________
How many hours per month are spent in temporal bone didactic education?	__________________________________
How often is your temporal bone didactics held relative to dissection labs?	◯ Less frequently than dissection labs<br>◯ As frequent as dissection labs<br>◯ More frequently than dissection labs
To what extent do Neurotology fellows at your program participate in temporal bone didactic education?	◯ Provide sole didactics education to residents<br>◯ Provide didactics education to residents alongside faculty<br>◯ Participate in didactics education alongside residents<br>◯ Both participate in and provide didactics education<br>◯ None of the above
To what extent do Otology fellows at your program participate in temporal bone didactic education?	◯ Provide sole didactics education to residents<br>◯ Provide didactics education to residents alongside faculty<br>◯ Participate in didactics education alongside residents<br>◯ Both participate in and provide didactics education<br>◯ None of the above
