# Supplementary material for: Current State of Temporal Bone Education: National Survey of U.S. Otolaryngology Residency Programs
Source: Laryngoscope. 2026 Apr 2;136(9):3966–72. doi: 10.1002/lary.70545 (PMC13460714; doi:10.1002/lary.70545)
Supplement: Supplementary file 2 [file LARY-136-3966-s002.pdf]

Temporal Bone Course

When: 3RD Friday
7:00 - 8:00 am LECTURE, Classroom C SOM
8:00 - 10:00 am LAB, 3R295A

1. This is a 4-year Temporal Bone Course schedule
2. Dissection assignments will be the same every year

SCHEDULE A

Session #	Date	4th Edition Reading Assignment	Lecture Topic	Dissection Assignment
1A	Sep 16	Ch 1 & 8	**Chronic Otitis Media I**	Canal wall up mastoidectomy, endolymphatic sac decompression, and ossicular chain reconstruction
2A	Oct 21	Ch 12 & 15	**Chronic Otitis Media II**	Facial recess and transmastoid facial nerve decompression
3A	Nov 18	Ch 34 & 36	**Surgery for Vertigo**	Translabyrinthine VIII nerve section
4A	Dec 16	Ch 27 & 28	**Acute Facial Paralysis**	Middle fossa VIII nerve section (new bone)
5A	Jan 20	Ch 20 & 21	**Otosclerosis**	Stapedectomy [*see below] and canal wall down mastoidectomy (old bone)
6A	Feb 17	Ch 18 & 31	**Management of the Complications of Mastoid Surgery**	Canal wall up mastoid and endolymphatic sac decompression
7A	Mar 17	Ch 30 & 58	**Cochlear Implants**	Extended facial recess, transmastoid facial nerve decompression, and lateral temporal bone
8A	Apr 21	Ch 44 & 52	**Tinnitus**	Approach to the jugular foramen and transcochlear approach
	**May 19**		**PRACTICUM**	

***At the conclusion of the Middle Fossa dissection, place a mineral-soaked cotton ball (upper cabinet to left of sink) in the EAC, to soften the canal skin. University Chief Resident to bring Skeeter Drill from OR.**

Temporal Bone Course

When: 7:00 - 8:00 am LECTURE

8:00 - 9:00 am LAB

1. This is now a 4-year Temporal Bone Course schedule
2. Added C and D with reading assignments and lecture topics
3. Dissection assignments will be the same every year, as always

SCHEDULE B

Session #	Date	Reading Assignment Otologic Surgery	Lecture Topic	Dissection Assignment
1B	9/20	Ch 9 & 11	**Chronic Otitis Media I**	Canal wall up mastoidectomy, endolymphatic sac decompression and ossicular chain
2B	10/18	Ch 16 & 17	**Chronic Otitis Media II**	Facial recess and transmastoid facial nerve decompression
3B	11/15	Ch 38 & 39	**Benign Positional Vertigo**	Translabyrinthine VIII nerve section
4B	12/20	Ch 29 & 57	**Facial Nerve Tumors**	Middle fossa VIII nerve section (new bone)
5B	1/17	Ch 14 & 56	**Complications of Chronic Otitis Media**	Canal wall up mastoid, and endolymphatic sac decompression
6B	2/21	Ch 22 & 55	**Medical Treatment of Otosclerosis**	Stapedectomy [*see below] and canal wall down mastoidectomy (old bone)
7B	3/20	Ch 4 & 5	**Pediatric Otology**	Extended facial recess, transmastoid facial nerve decompression, and lateral temporal
8B	4/17	Ch 26 & 53	**Miscellaneous Disorders** (exostosis, osteomas, serous mastoiditis, idiopathic hemotympanum, abnormally patent eustachian tube)	Approach to the jugular foramen and transcochlear approach

***At the conclusion of the Middle Fossa dissection, place a mineral-soaked cotton ball (upper cabinet to left of sink) in the EAC, to soften the canal skin. University Chief Resident to bring Skeeter Drill from OR.**

Temporal Bone Course

When: 7:00 - 8:00 am LECTURE

8:00 - 10:00 am LAB

1. This is now a 4-year Temporal Bone Course schedule
2. Added C and D with reading assignments and lecture topics
3. Dissection assignments will be the same every year, as always

SCHEDULE C

Session #	Date	Reading Assignment Otologic Surgery	Lecture Topic	Dissection Assignment
1C	Oct 21	Ch 11 & 40	**Chronic Otitis Media I**	Canal wall up mastoidectomy, endolymphatic sac decompression, and ossicular chain reconstruction
2C	Nov 18	Ch 64 & 65	**Chronic Otitis Media II**	Facial recess and transmastoid facial nerve decompression
3C	Dec 16	Ch 42 & 35	**Superior Semicircular Canal Dehiscence**	Translabyrinthine VIII nerve section
4C	Jan 20	Ch 48, 49, 50	**Acoustic Tumors**	Middle fossa VIII nerve section (new bone)
5C	Feb 17	Ch 24 & 27	**Revision Otosclerosis Surgery**	Stapedectomy [*see below] and canal wall down mastoidectomy (old bone)
6C	Mar 17	Ch 20 & 60	**Spontaneous Cerebrospinal Fluid Leaks and Encephaloceles**	Canal wall up mastoid and endolymphatic sac decompression
7C	Apr 14	Ch 8 & 54	**Office Management of the Draining Ear**	Extended facial recess, transmastoid facial nerve decompression, and lateral temporal bone
8C	May 19	Ch 45 & 51	**Petrous Apex Lesions**	Approach to the jugular foramen and transcochlear approach

***At the conclusion of the Middle Fossa dissection, place a mineral-soaked cotton ball (upper cabinet to left of sink) in the EAC, to soften the canal skin. University Chief Resident to bring Skeeter Drill from OR.**

Temporal Bone Course

When: 7:00 - 8:00 am LECTURE, Classroom C SOM

8:00 - 10:00 am LAB, 3R295A

1. This is now a 4-year Temporal Bone Course schedule
2. Added C and D with reading assignments and lecture topics
3. Dissection assignments will be the same every year, as always

SCHEDULE D

Session #	Date	Reading Assignment Otologic Surgery	Lecture Topic	Dissection Assignment
1D	Sep 17	Ch 13 & 47	**Chronic Otitis Media I**	Canal wall up mastoidectomy, endolymphatic sac decompression, and ossicular chain reconstruction
2D	Oct 15	Ch 59 & 65	**Chronic Otitis Media II**	Facial recess and transmastoid facial nerve decompression
3D	Nov 19	Ch 37 & 41	**Medical Treatment of Dizziness**	Translabyrinthine VIII nerve section
4D	Dec 17	Ch 43 & 46	**Other Skull Base Tumors**	Middle fossa VIII nerve section (new bone)
5D	Jan 21	Ch 24 & 25	**Otosclerosis: Complications and Special Problems**	Stapedectomy [*see below] and canal wall down mastoidectomy (old bone)
6D	Feb 18	Ch 61 & 62	**Facial Reanimation and Eye Care**	Canal wall up mastoid and endolymphatic sac decompression
7D	Mar 18	Ch 32 & 33	**Implantable Hearing Aids and BAHA**	Extended facial recess, transmastoid facial nerve decompression, and lateral temporal bone
8D	Apr 15	Ch 53	**Temporal Bone Malignancies**	Approach to the jugular foramen and transcochlear approach

***At the conclusion of the Middle Fossa dissection, place a mineral-soaked cotton ball (upper cabinet to left of sink) in the EAC, to soften the canal skin. University Chief Resident to bring Skeeter Drill from OR.**
